# Investigation of hub genes involved in Turner syndrome using biological informatics methods

**DOI:** 10.1097/MD.0000000000029069

**Published:** 2022-03-18

**Authors:** Tiantian Cheng, Xiaoli Li, Jinhu Chen, Linlin Yang, Jing Liu, Guangyao Song, Huijuan Ma

**Affiliations:** ^a^ *Department of Internal Medicine, School of Clinical Medicine, North China University of Science and Technology, Tangshan, Hebei, China.,* ^b^ *Department of* *Endocrinology and Metabolic Diseases, Hebei General Hospital, Shijiazhuang, Hebei, China.,* ^c^ *Department of Internal Medicine, Hebei Medical University, Shijiazhuang,* *Hebei, China.,* ^d^ *Hebei Key Laboratory of Metabolic Diseases, Hebei General Hospital, Shijiazhuang, Hebei, China.*

**Keywords:** bioinformatics analysis, differentially expressed genes, hub gene, microarray analysis, Turner syndrome

## Abstract

**Background:**

This study aimed to explore candidate genes and their potential interaction mechanism critical to the pathophysiology of Turner syndrome by using the Gene Expression Omnibus database.

**Methods:**

GSE58435 data set was obtained by querying the Gene Expression Omnibus database. Differentially expressed genes (DEGs) were screened using R and subsequently annotated by Gene Ontology. Functional enrichment analysis was performed based on the Kyoto Encyclopedia of Genes and Genomes database for annotation, visualization, and integrated discovery. A protein-protein interaction network of different genes was constructed based on the STRING database, in which hub genes were explored through Cytoscape software. The expression of the hub genes was verified by analyzing the gene expression in the GSE46687 data set.

**Results:**

A total of 733 differential genes were identified. These differentially expressed genes were significantly enriched in nucleoplasm and nucleus. Their molecular function was concentrated on DNA binding and transcription, coronary artery, and adipose tissue development. According to the annotation of Kyoto Encyclopedia of Genes and Genomes, the identified DEGs were mainly enriched in inflammatory mediator regulation of TRP channels, osteoclast differentiation. A total of 10 hub genes (HIST1H2BA, TRIM71, HIST1H2BB, HIST1H4D, TNF, TP53BP1, CDCA8, EGF, HMG20B, and BCL9) were identified from the constructed protein-protein interaction network. These genes were discovered to be highly expressed in osteoclasts, ovaries, digestive tract, blood, and lymphatic tissues through the online application of human protein atlas.

**Conclusion:**

In this study, 733 DEGs and 10 hub genes were identified. They would be new candidate targets in Turner syndrome.

## 1. Introduction

Turner syndrome (TS) is a chromosomal disorder in women characterized by complete of partial loss of 1 of the 2 X-chromosomes. Its incidence is 3 to 5 in 10,000 new-born girls. It is characterized by small stature, absent menarche, and absent growth spurt. Other clinical features of TS may include typical dysmorphic stigmata, renal, cardiac, skeletal, and metabolic abnormalities.^[1]^ It also involves gastrointestinal symptoms like, for example, celiac disease (CD).^[2]^ Half of the children with TS have congenital heart abnormality, like mitral stenosis and aortic stenosis.^[3,4]^ Patients also develop liver disease at adult age.^[5]^

Microarray technology and bioinformatics analysis allow for the screening of genetic changes at the genome level. In this study, statistical analysis and data mining techniques were employed to identify novel candidate targets with high specificity and sensitivity by detecting genes with significant differences in amniotic fluid expression between TS and normal samples. Our findings contribute to the understanding of the genetic etiology of TS and provide a new perspective for clinical diagnosis and treatment.

We present the following article in accordance with the MDAR reporting checklist.

## 2. Materials and methods

### 2.1. Microarray data and preprocessing

The microarray dataset GSE58435 deposited by Massingham needs to be downloaded from Gene Expression Omnibus (GEO, https://www.ncbi.nlm.nih.gov/geo/). This data set is based on the GPL570 human genome U133 Plus 2.0 array platform. There are 10 samples included in this experiment: 5 TS samples and 5 normal samples. These samples are all obtained from amniotic fluid. The gene expression data in this chip were extracted for further analysis. Additionally, the annotation file of GPL570 is also downloaded from GEO. The study was conducted in accordance with the Declaration of Helsinki (as revised in 2013).

### 2.2. Data quality evaluation and data processing

R affyPLM library (version 4.0.4) was used to perform regression calculations on the data set. Besides, the affy libraries are adopted to obtain RNA degradation data, and the data quality is further evaluated. The corresponding graphics are drawn to make the data quality more intuitive.

The affy package is employed to correct and standardize the background of the TS group and normal group and thus eliminate the errors caused by non-experimental factors. Then, the corrected data of the 2 groups are combined and summarized to obtain a corrected complete data matrix. The limma libraries were utilized to screen the differential expression genes with the detect conditions of difference multiple >2 times and *P* < .05 (logfoldchange > 1 or logfc < [-1] and adjustment *P* < .05). The qualified differentially expressed genes (DEGs) in TS and normal chips were obtained. Specifically, logFC < 0 indicates that the gene expression is down-regulated: logFC > 0 suggests that gene expression is up-regulated. According to the detected differential expression genes, the volcano map and heat map are drawn using R-related visualization functions.

### 2.3. Functional enrichment analysis of DEGs

Annotation, visualization, and integrated discovery database (DAVID) are commonly used tools in bioinformatics research. In this study, the DAVID 6.8 was employed to annotate Gene Ontology (GO) function and analyze Kyoto Encyclopedia of Genes and Genomes (KEGG) function enrichment of different genes. GO analysis, involving biological process (BP), cellular component (CC), and molecular function (MF), was conducted to predict protein function. Moreover, the ggplot2 package of R was used to visualize the screened data and make a bar plot and circle chart.

Additionally, the differential genes detected above were imported into DAVID 6.8 online analysis website for KEGG signaling pathway. KEGG pathway analysis was performed to assign a series of DEGs to specific pathways, so as to build a network of interactions, reactions, and relationships among molecules. The ggplot2 package of R was taken to make bubble charts and visualize each pathway.

### 2.4. Construction of protein-protein interaction between DEGs

STRING (https://string-db.org/) is an online database of known and predicted protein-protein interactions (PPI). These interactions include physical and functional associations. The data were primarily obtained from computational prediction, highthroughput experiments, automatic text mining, and co-expression networks. The data screened by high-throughput experiments were imported into the online application of STRING, and >0.7 was set as the threshold. If the comprehensive score is >0.7, these DEGs are considered essential protein pairs. The protein pairs were further sorted and analyzed using the cytoscapehubba of Cytoscape v3.9.0. The top 10 hub genes were detected. Then, the online application of the human protein atlas was performed to analyze the tissue-specific expression of these 10 genes.

### 2.5. Verification of hub genes

The microarray expression profiling data set GSE46687 was downloaded from gene expression omnibus. There were 36 samples in this experiment: 26 TS patients and 10 normal children. The samples were obtained from children’s blood. Through the GEO2R analysis of online database, the differential genes were screened out, and the screening conditions were set as difference multiple >2 times and *P* < .05 (logfoldchange > 1 or logfc < [-1] and adjp < 0.05). The hub genes screened in the GSE58435 database were searched from the screened differential genes.

## 3. Results

### *3.1. Quality evaluation of TS and normal data sets and the* *DEG*

The quality of the data set was visualized with R. A quality evaluation map is illustrated in Fig. 1. Figure 1A presents the relative logarithmic expression. There are 10 samples in a horizontal baseline, suggesting that the data set is of good quality. Figure 1B is a graph of RNA degradation. The fluorescence intensity at the 5′ end of the chip is much lower than that at the 3′ end. Thus, the abscissa runs from left to right from 5′ end to 3′ end. The slope in the graph also demonstrates the high quality of the data.

**Figure:**
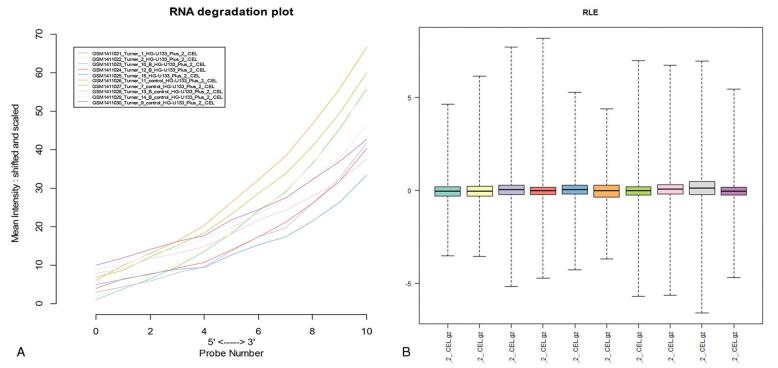
**Figure 1.** Data set quality evaluation results. (A) Relative logarithmic expression (RLE), which is the logarithm of the expression value of a probe group in a certain sample divided by the median value expressed by the probe group in all samples, reflecting the consistency of parallel experiments. (B) RNA degradation diagram, with different colors representing 10 samples.

The limma package and impute package in R were used to detect differential expression genes, with the detecting conditions of logfoldchange > 1 or logfoldchange < (-1), and adjp < 0.05. Besides, 733 significantly differentially expressed genes were obtained. Compared with normal samples, the number of genes up-regulated and down-regulated in TS was 387 and 346, respectively. The volcano and heat map of the detected differential genes is exhibited in Fig. 2.

**Figure:**
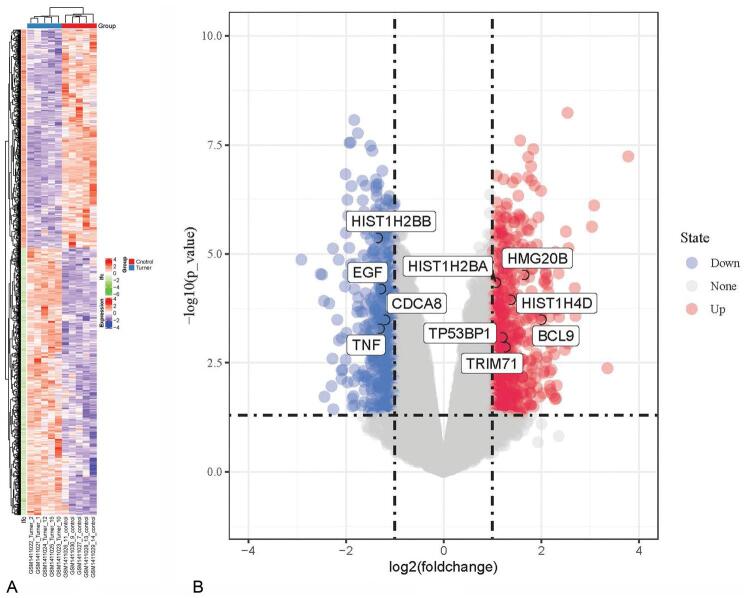
**Figure 2.** Processing result of data set. (A) The heat map shows 733 genes with the most significant differences. Red represents a high expression signal, and blue represents a low expression signal. (B) Volcano map, showing the DEGs in the chip compared with normal amniotic fluid; red dots represent Turner highly expressed genes, and blue dots represent Turner low expressed genes. DEGs = differentially expressed genes.

### *3.2. Enrichment analysis and signal pathway analysis of* *DEGs*

The GO analysis of DEGs was conducted with DAVID 6.8 online tool, and 733 differential genes were further studied. The GO analysis involves BP, CC, and MF. Accordingly, the detected differential expression genes are imported into DAVID. Then, the detected results are taken as *P* < .05, and 27 terms are obtained. The barplot is obtained by the R ggplot2 library (Fig. 3A), and the first 10 terms are screened out. Moreover, a circle diagram is made through the GOplot package (Fig. 3B). The results revealed that the most abundant terms include GO:0006355, GO:0006351, GO:0005634, GO:0046872, GO:0003677, GO:0003700, and GO:0003676. Hence, the genes in CC mainly involve the nucleus, nucleoplasm. MF indicates that the main functions of these genes are to regulate the DNA binding, transcription factor activity, nucleic acid binding, arylesterase activity. BP analysis demonstrated that the functions of these genes are mainly engaged in the following biological processes: the regulation of transcription, DNA-templated transcription, positive regulation of tyrosine phosphorylation of STAT3 protein, coronary vasculature development, adipose tissue development, transcription from RNA polymerase II promoter, and negative regulation of intracellular estrogen receptor signaling pathway (Table 1). Additionally, the data are visualized through the GOplot package and ggplot2 package of R software. In this study, a chart of the terms obtained from the analysis of all the screened DEGs is acquired while the up-regulated and downregulated genes are analyzed. Besides, *P* < .05 is taken to visualize the results (Fig. 3C and D). In Fig. 3C and D, the qualified GO enrichment analysis results of up-regulated genes and downregulated genes contain 20 items and 17 items, respectively. It was revealed by analyzing the data in detail that the obtained data is not the sum of simple up-regulation genes and down-regulation genes, though most enrichment results are the same in the process of GO analysis. For example, in addition to the above biological processes, BP also includes protein glycosylation. The enrichment analysis of down-regulated genes also yielded similar results.

**Table 1:** Differential genes between Turner group and normal group were screened by using the limma package in R language.

**Turner vs normal**	**Genesymbol**
Up regulated genes (387)	POTEM, LRRC56, LOC101928725, TMEM120A, LOC100506691, WFDC13, LOC101928571, NMU, LOC101928707, NECAB2, GNGT1, LOC100133130, LOXHD1, TPRA1, LOC100505715, NKRF, LINC00582, LOC101928865, TPBGL, ZNF155, SPRY3, TOR4A, CCNJL, C7orf62, SARNP, CRTC2, SLC41A1, HDAC5, CNRIP1, ZNF780B, CPLX1, LOC100289361, SMCR5, TEKT3, EMC6, RNF175, TRUB2, CARM1, PADI1, LURAP1L, TTLL12, C1orf192, NARFL, LOC253044, SHANK2-AS3, ZNF625, CXorf31, PLB1, ABCG4, CST2, PFKFB4, PCDHB12, MAD2L1BP, LOC100507336, LOC102724640, FLJ25917, LINC01356, LOC729296, PARP1, S100A12, CCNF, IGFBPL1, LOC102725454, KIRREL3, IKZF5, ZNF2, ZNF559, HNRNPUL2, GOLGA4, C14orf132, ZNF35, LINC01304, PIWIL1, ARHGAP31, RUSC2, TRIM47, CMTR1, CD27, POLDIP3, PARP14, KBTBD2, DNAJB11, MFSD5, NOL10, SIT1, HMBS, DEPDC1B, PTPN23, LRFN3, LEPREL4, ZNF175, CDON, TLR5, NDUFV2-AS1, COQ10A, VPREB3, IER2, TPPP, ZNF823, STK16, MDFI, AGAP11, HMG20B, CAAP1, GAK, MC3R, FDXR, EMR2, NACC1, ESRRG, C17orf58, PROCR, ZDHHC17, KIAA0586, EPB41L4A-AS2, LOC100630918, MUTYH, HIST1H2BA, CYP7B1, CTSW, ANKRD52, GPKOW, ZBTB3, PRAMEF12, LOC101929488, COPA, IVL, SMIM6, APOBR, ZNF516, ATP6AP1, FAM219A, PRSS21, DENND4A, NAT2, CWF19L1, ZNF304, BTG2, HES5, FAM178A, ARNTL2-AS1, SCMH1, LOC100506119, KLHL9, DENND1C, RPRD1B, HIST1H4D, CARD6, SPRR2C, MPI, MFAP4, MAP2K6, SHROOM2, RASL12, KIR2DL5A, SACS, LOC101929177, NRCAM, LOC101927420, ORC5, IFIT2, ZNF142, ATP6V1A, SLCO2A1, GUCY2D, NOSTRIN, BATF, LOC101927839, GCNT1, PRKXP1, CASC7, SPIB, HTRA2, KDM1B, SPG21, LAMC2, C2CD2, ATP5A1, RPTOR, SLC9A8, TUBG1, FAM106A, BSN, DDRGK1, GMDS, GPR126, APOBEC3C, KCNJ2, GMPR, ZBED6, ENO2, DEF6, ATG14, LINC00520, CYTH2, BCL9, ZSCAN31, IGFBP5, PIGC, THEM6, POLR2J2, FYCO1, FBN3, PRR22, FNDC8, FAM166B, FLJ45482, FOXP4, SEC23A, VEGFB, CHIAP2, B3GAT1, ATG12, GLRX2, GGT5, ZNF770, BDKRB1, SPDL1, STIL, TMEM87B, SNTA1, LOC375196, LRRC8E, SERPINB9P1, MYRFL, ZNF253, PKD2L2, EIF3J-AS1, SH2B2, PLEKHG4B, TIMP3, FBXO30, TSHZ3, SEMA4B, BSPRY, ZNF8, PTDSS2, ZUFSP, IP6K3, ELP5, TP53BP1, KLF11, FAM217B, IL1R1, C19orf82, SLC25A1, TMEM57, S100A2, ZSCAN21, RNF44, GALNT2, KATNAL1, DIABLO, GINS3, F2RL1, FUT10, CHUK, FAM175B, FOXRED1, SLC41A3, LOC100129550, BREA2, NIPSNAP1, PMPCA, DERL2, TAB1, DYNC1LI1, TMEM159, TCF20, RGAG4, CCDC88B, C9orf40, TRIM71, MYO16, RPP38, ZFYVE27, SOX12, GPATCH11, LEP, UBXN7, GEMIN2, DAXX, MED25, LOC101928635, DCAF16, LOC100288570, BCAM, TMEM194B, TCEA3, BIK, TRIB2, ZNF844, LOC100131170, MPLKIP, ZNF275, CRISP3, PRICKLE1, PROS1, OSBPL8, ZNF318, PCDHGB8P, HYAL1, C4orf36, VPS37B, MCEE, PARP12, LOC100652824, RPS29, PMEPA1, KLK13, KEAP1, LOC101928424, RBP7, UBL7-AS1, UBE2E3, KDELC2, FHL2, NSL1, WDR3, IGF2R, PXN, ATN1, TLCD1, ZNF639, WBSCR17, MTMR3, TSHZ1, HMGN1, CA11, LSP1, CLDN12, CALCOCO1, HCCS, C1orf226, HIST1H2BJ, ZNF132, LOC101927783, P2RY2, MAPK9, GNRHR2, RFX7, ANP32D, VSNL1, B4GALT5, PIGH, PRICKLE3, ZNF418, SSUH2, KRT24, RNF126P1, BCAP31, EP300-AS1, TMEM97, POLR3F, TADA2A, ZNF595, SPRTN, HECTD3, STAMBP, HERC1, NSRP1, WASF2, ANG, PHF13, LRP2BP, ALDH3A2, C1orf216, FURIN, ASPSCR1, PITX2, LOC283454, RGL1, SMAD5, IQUB, KRT15, FAM171A1, OR7E14P, NBEA, PRR15, ZNF236, STX5, SDF2, SLC26A11, ZBED9, B4GALT2, MAF1, THOC1
Down regulated genes (346)	C5orf49, CLDN20, SULT4A1, LOC101927811, KRTAP5-9, LOC101928580, PLA2G4D, LOC221272, SOX8, CLCN2, LOC101928152, RAMP3, LOH12CR2, C22orf39, SCG2, FLJ90680, LOC101929465, CLDN10-AS1, KLRC3, DHCR7, ZNF366, HLA-DMB, REG1A, SH2D4B, LOC101928729, LOC100422781, IL20, SLFN11, TRDV3, CCDC148-AS1, UGT1A1, TMEM55A, DPYS, CD33, LINC01204, LOC101927592, RFPL2, LOC151484, ADPGK, LOC101927720, LINC00708, IL25, ZP2, KLF15, AQP11, GFI1B, SPA17, RAD54L, TKTL2, LOC728073, C6orf99, KLRB1, RAB40A, NACA2, DYDC1, HCRT, HIST1H2BB, FAM24A, LOC340085, SMIM10, CXCL3, CASP12, ONECUT1, DPP3, LOC257152, C7orf73, KRT32, LOC642533, DIRC3, OLR1, USP27X-AS1, SGCZ, LOC100996255, TCFL5, GTSF1, FDXACB1, RNLS, ATP6V0E2-AS1, REC114, S100A13, CRYGB, C11orf84, FBXO16, WDR18, FLJ22763, SMYD3, CLDN2, CDX2, NPIPB15, C1orf158, KHDRBS2, LOC202025, LOC101927206, KRTAP4-11, LOC100131532, MEF2BNB, SCNN1D, STRIP1, FLT3, TTC4, LOC221946, P2RX5, CORO7, FAM149A, AFAP1L1, GDF3, FOXJ3, POM121L12, WDYHV1, RARS, THTPA, SPAG5-AS1, PTPDC1, LOC100287210, CLCN6, RBPJL, BPIFB4, ZNF835, LOC100506790, PSTPIP2, DHCR24, SV2A, MASTL, ZNF324, TMPO-AS1, COQ6, ZNF385D, EGF, BATF2, C19orf57, FAM3B, ROCK2, IL21, YEATS2, ALMS1P, B9D2, SHMT1, ERMN, CHRDL1, LOC286087, TMEM143, SLC4A1, YARS2, GADL1, APOL5, FAM187B, LOC100507564, TIGD3, LOC100507480, SLC25A16, SOCS2-AS1, CCR9, TLX3, LSM10, MIATNB, TTPA, CFHR2, LOC100506469, CHTF18, TPST1, RNASEH1-AS1, SLC45A4, CRLF2, LOC100506406, LOC100132207, MST1, ADCY6, RASSF1, TRPV3, LOC284865, ATP13A1, LOC101929224, LGALS14, VANGL1, GAL3ST3, PXYLP1, GCFC2, C9orf53, LOC101927609, FAM117A, SCRN2, ERC2, XPNPEP2, CNNM3, FGF9, C10orf91, LOC101927164, PCP4, C19orf60, ZNF140, ZNF70, SLC27A6, MLF1, C10orf71, SBF2-AS1, ZNF14, COMMD10, TWISTNB, ADARB2, KLF17, MAP1LC3B2, COG7, LOC100507065, CDCA8, C15orf52, MAGEF1, PTGFRN, TTTY10, LOC100506047, TBCCD1, HAPLN3, PRR13, HS3ST6, MYL12B, RAB28, CSTF2, WDPCP, C3P1, ACADM, NDUFS7, CA1, NUPL2, KPTN, LENEP, ZNF793, KCNQ1DN, PGLYRP2, CLEC2D, LOC400541, TNF, PHC2, ZNF782, LOC101927248, STAT4, CHCHD4, TIGD2, GEN1, IPO4, ODF2, LRRC47, ACVR1, ZNF214, HPS3, ISL1, FAM155A, HCAR3, METTL1, TUBB2B, PTK6, OR1I1, NUDT6, LOC400622, C2orf81, THAP10, CD248, KIAA0391, GPCPD1, LOC101927550, LOC100132735, TAF2, TMEM117, NOB1, LOC102724975, GPN1, APMAP, SCUBE2, C7orf55, RRP36, LOC100129603, SLC25A26, PRR12, SOX18, NUDT16L1, CREB3L3, EBLN2, FBLN2, PIP4K2C, TRPC4AP, LOC339988, COX11, PTPRH, NHLRC4, ZDHHC6, CAMKV, DPY19L2P4, IFI27, GPC3, OXER1, SPI1, LOC100507291, ADCK1, LYPD5, DDX10, ZNF211, ZNF423, PRRG1, LOC100506922, NKX6-1, TRPM5, ZNF280A, INPP5J, CEP55, EFHD1, ZNF320, ZNF331, ISX, HNRNPU-AS1, ISOC1, TUBAL3, ARHGAP12, PDIK1L, BRI3BP, PMEL, IRF7, TRIM78P, RPA2, PLOD3, PON3, PRKCDBP, ENTPD7, MED14OS, DSEL, CDX1, TULP3, KCNQ1-AS1, FIBP, RGS7BP, ZSCAN12P1, TEX261, FLG, LOC253805, DDX19B, SPAG5, CCRN4L, RASSF9, LHPP, GGTLC1, OR7E37P, ADHFE1, GRAMD1B, GTF2E1, ACAT1, FMO4, ATP6V1C2, STK36, ODF3L1

**Figure:**
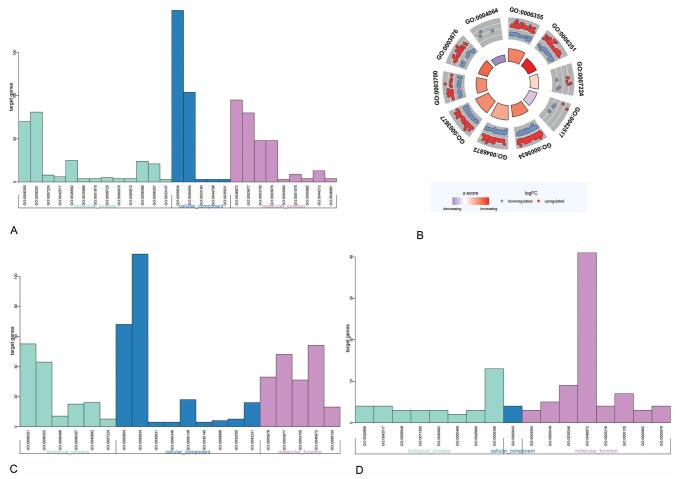
**Figure 3.** GO enrichment analysis results. (A) From the enrichment analysis results of all DEGs, terms were obtained by taking *P* < .05. (B) For GO enrichment analysis of all differential genes, the first 10 GO data items are selected as visual circles. Red dots indicate up-regulated genes, and blue dots indicate downregulated genes. The closer the inner circle is to the outer circle, the more genes there are in this item. The darker the inner circle, the more up-regulated genes account for this item. (C) The result of up-regulating DEGs. (D) The result of down-regulating DEGs. DEGs = differentially expressed genes, GO = Gene Ontology.

The KEGG pathway analysis was performed to verify DEG function and signal pathway enrichment. A total of 11 essential signal paths have been identified. Similarly, the results of KEGG path analysis are visualized through the application package of R software (Fig. 4). Figure 4A indicates that the pathways with the most gene enrichment include inflammatory mediator regulation of TRP channels; others involve osteoclast differentiation, TNF signaling pathway, toxoplasmosis, mucin-type O-glycan biosynthesis, beta-Alanine metabolism, and inflammatory bowel disease (IBD) (Table 2). The separate analysis of the up-regulated and down-regulated genes demonstrated the epithelial cell signaling in *Helicobacter pylori* infection in the KEGG pathway analysis of up-regulated genes. Moreover, down-regulated genes may be engaged in the formation of hepatitis B and C.

**Table 2:** GO enrichment analysis of DEGs.

**Category**	**Term**	**Description**	**Count**	***P*** **value**
GOTERM_BP_DIRECT	GO:0006355	Regulation of transcription, DNA-templated	70	3.21E-04
GOTERM_BP_DIRECT	GO:0006351	Transcription, DNA-templated	81	.003068
GOTERM_BP_DIRECT	GO:0007224	Smoothened signaling pathway	8	.004809
GOTERM_BP_DIRECT	GO:0042517	Positive regulation of tyrosine phosphorylation of Stat3 protein	6	.005515
GOTERM_BP_DIRECT	GO:0045892	Negative regulation of transcription, DNA-templated	25	.01806
GOTERM_BP_DIRECT	GO:0030866	Cortical actin cytoskeleton organization	4	.034939
GOTERM_BP_DIRECT	GO:0001819	Positive regulation of cytokine production	4	.034939
GOTERM_BP_DIRECT	GO:0009725	Response to hormone	5	.037698
GOTERM_BP_DIRECT	GO:0060976	Coronary vasculature development	4	.038836
GOTERM_BP_DIRECT	GO:0060612	Adipose tissue development	4	.038836
GOTERM_BP_DIRECT	GO:0006366	Transcription from RNA polymerase II promoter	24	.040327
GOTERM_BP_DIRECT	GO:0006357	Regulation of transcription from RNA polymerase II promoter	21	.047932
GOTERM_BP_DIRECT	GO:0033147	Negative regulation of intracellular estrogen receptor signaling pathway	3	.049505
GOTERM_CC_DIRECT	GO:0005634	Nucleus	199	6.01E-04
GOTERM_CC_DIRECT	GO:0005654	Nucleoplasm	104	.013486
GOTERM_CC_DIRECT	GO:0033180	Proton-transporting V-type ATPase, V1 domain	3	.022409
GOTERM_CC_DIRECT	GO:0044798	Nuclear transcription factor complex	3	.041477
GOTERM_CC_DIRECT	GO:0035631	CD40 receptor complex	3	.041477
GOTERM_MF_DIRECT	GO:0046872	Metal ion binding	95	1.03E-04
GOTERM_MF_DIRECT	GO:0003677	DNA binding	80	1.20E-04
GOTERM_MF_DIRECT	GO:0003700	Transcription factor activity, sequence-specific DNA binding	48	.001525
GOTERM_MF_DIRECT	GO:0003676	Nucleic acid binding	48	.002499
GOTERM_MF_DIRECT	GO:0004064	Arylesterase activity	3	.013423
GOTERM_MF_DIRECT	GO:0001078	Transcriptional repressor activity, RNA polymerase II core promoter proximal region sequence-specific binding	9	.022957
GOTERM_MF_DIRECT	GO:0000982	Transcription factor activity, RNA polymerase II core promoter proximal region sequence-specific binding	4	.033694
GOTERM_MF_DIRECT	GO:0044212	Transcription regulatory region DNA binding	13	.035543
GOTERM_MF_DIRECT	GO:0046961	Proton-transporting ATPase activity, rotational mechanism	4	.046203

**Figure:**
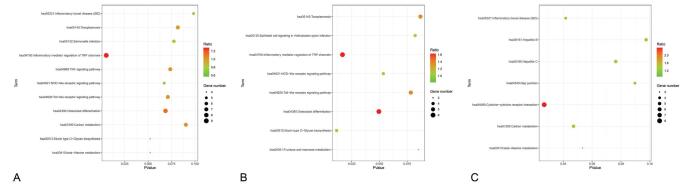
**Figure 4.** KEGG visualization results. The abscissa represents the *P*-value, the ordinate represents different paths. The larger the dots in the figure, the more genes contained in this pathway; the redder the dot color, the higher the probability of genes rich in this pathway. (A) Results of KEGG enrichment analysis of all DEGs. (B) Enrichment analysis results of up-regulated DEG. (C) Enrichment analysis results of down-regulated DEGs. DEGs = differentially expressed genes, KEGG = Kyoto Encyclopedia of Genes and Genomes.

### 3.3. PPI network analysis of DEGs

The PPI network is constructed using the online analysis website STRING, and the data are further analyzed for protein interaction. The differential genes are imported into the online analysis website, and the visualization graph is composed of 388 edges and 608 nodes.

In this paper, cytohubba in Cytoscape is employed to calculate the hub genes in 12 ways. Then, the central elements in complex networks by intersection are discovered, such as HIST1H2BA, TRIM71, HIST1H2BB, HIST1H4D, TNF, TP53BP1, CDCA8, EGF, HMG20B, and BCL9. According to the results of functional enrichment analysis, these 10 genes are mainly involved in DNA binding, DNA transcription, nucleus, coronary vessels, and neural tube development (Table 3).

**Table 3:** KEGG pathway enrichment analysis of DEGs.

**Term**	**Description**	**Count**
hsa04750	Inflammatory mediator regulation of TRP channels	9
hsa00512	Mucin type O-Glycan biosynthesis	4
hsa00410	beta-Alanine metabolism	4
hsa04621	NOD-like receptor signaling pathway	5
hsa04380	Osteoclast differentiation	8
hsa04620	Toll-like receptor signaling pathway	7
hsa04668	TNF signaling pathway	7
hsa05132	Salmonella infection	6
hsa05145	Toxoplasmosis	7
hsa01200	Carbon metabolism	7
hsa05321	Inflammatory bowel disease (IBD)	5
DEGs = differentially expressed genes, KEGG = Kyoto Encyclopedia of Genes and Genomes		

Human protein atlas (https://www.proteinatlas.org/) online is also used to analyze the tissue-specific expression of the selected genes. The results revealed that HIST1H2BB, HIST1H2BA, HIST1H4D, TNF, CDCA8, and HMG20B were mainly engaged in the expression of osteoclasts. Besides, BCL9 was highly expressed in the ovary; TRIM71 was highly expressed in lung and brain; TP53BP1 was mainly expressed in thyroid tissue; EGF was expressed in kidney, pancreas, and muscle; TP53BP1, CDCA8, and HMG20B were highly expressed in the digestive tract; TNF, CDCA8, and HMG20B were highly expressed in blood; HIST1H4D was also expressed in lymphoid tissue.

### 3.4. Verification analysis

The network online database GEO2R was utilized to analyze the GSE46687 data set. The differential genes were selected and compared with the key genes selected from the GSE58435 database. The expression of HIST1H4D, TNF, TP53BP1, CDCA8, EGF, HMG20B, and BCL9 in the GSE46687 data set was significant and consistent (Table 4).

**Table 4:** Genes of interest.

	**Genes**	**Description**	***P*** **value**	**logFC**
Up regulated genes	HIST1H2BA	DNA binding	4.51E-05	1.069439
	TRIM71	neural tube development	.001371	1.267882
	HIST1H4D	DNA binding	.000111	1.372507
	TP53BP1	DNA binding	.000818	1.202516
	HMG20B	DNA binding	2.98E-05	1.648255
	BCL9	Nucleus	.000319	2.002039
Down regulated genes	HIST1H2BB	DNA binding	4.23E-06	-1.35259
	TNF	Negative regulation of transcription, DNA-templated	.000513	-1.31322
	CDCA8	Nucleus	.000323	-1.19809
	EGF	Coronary vasculature development	6.28E-05	-1.28447

## 4. Discussion

TS is a sex chromosome genetic syndrome with a variety of clinical symptoms. There is little research on the molecular level of TS. The research sample is amniotic fluid, and only GSE58435 is retrieved from the GEO database. In this study, 733 differential expression genes were detected by analyzing the gene expression microarray of 5 TS samples and 5 normal samples from GSE58435. In this study, several overlapping hub genes were discovered through the differential gene analysis of the GSE46687 data set to better verify the stability of our analysis results.

The GO functional annotation analysis revealed that the genes with significant differences in expression were mainly involved in the processes of DNA binding and transcription, regulation of protein tyrosine, production of cytokines, response to hormones, development of coronary artery, and adipose tissue. GO analysis showed that the genes were involved in DNA binding and transcription. DNA methylation changes were widespread on all autosomal chromosomes in 45,X and in 47,XXY individuals, with Turner individuals presenting 5 times more affected loci. Differentially methylated CpGs, in most cases, have intermediate methylation levels and tend to occur outside CpG islands, especially in individuals with Turner syndrome.^[6]^ In a large number of individuals, a study verified several loci by pyrosequencing and observed only weak inter-loci correlations between the verified regions. This suggests a certain stochastic/random contribution to the methylation changes at each locus.^[6]^ The research analyzed DNA methylation changes during reprogramming of male and female somatic cells and in resulting induced pluripotent stem cells (iPSCs). At an intermediate reprogramming stage, somatic and pluripotency enhancers are targeted for partial methylation and demethylation. Demethylation within pluripotency enhancers often occurs at ESC binding sites of pluripotency transcription factors. Late in reprogramming, global hypomethylation is induced in a female-specific manner. Genome-wide hypomethylation in female cells affects many genomic landmarks, including enhancers and imprint control regions, and accompanies the reactivation of the inactive X chromosome. The loss of 1 of the 2 X chromosomes in propagating female iPSCs is associated with genome-wide methylation gain.^[7]^ EGF plays a protective role in oxidative stress of the coronary artery. RNA molecular analysis was conducted on peripheral blood cells from patients with stable coronary artery disease; the mRNA levels of inflammation and oxidative stress markers, such as RORγt (T helper cell 17 cell marker), FoxP3 (regulatory T cell marker), NLRP3, ICAM1, SIRT1, Notch ligands JAG1 and DLL4, and HES1 (Notch target gene), were determined; the changes in SIRT1 and HES1 mRNA were related to serum epidermal growth factor.^[8]^ The EGF level was down-regulated in the key differential genes selected by us. The GO enrichment analysis also suggested that EGF was involved in the development of coronary vessels in TS patients. Magnetic resonance imaging 4-D flow-based examination of TS patients demonstrated that the diameter of the aorta was significantly larger than that of normal people, and the eddy current of ascending aorta increased, leading to the occurrence and development of aortic dissection.^[9]^ This aortic abnormality mainly involves the left filling artery, and the most crucial abnormality is the absence of the left aorta.^[10]^ Furthermore, the abnormality of the aorta is a risk factor of hypertension in normal people and TS patients.^[11]^ TRIM71 is also an important DEGs, and GO enrichment analysis shows that it is involved in the development of neural tube. Molecular studies on congenital hydrocephalus show that, exome sequencing of 125 CH trios and 52 additional probands identified 3 genes with significant burden of rare damaging de novo or transmitted mutations, in addition to TRIM71, there are SMARCC1 and PTCH1. Strikingly, all 3 genes are required for neural tube development and regulate ventricular zone neural stem cell fate.^[12]^

KEGG enrichment analysis suggested that many of these genes were associated with osteoclast differentiation, alanine metabolism, IBD, *H pylori* infection, hepatitis, and other signal pathways. Cui et al^[13]^ induced and cultured pluripotent stem cells (iPSC) of TS patients, discovering that osteoclasts were highly expressed in the TS population. In KEGG analysis, we revealed that TNF may be involved in the osteoclast differentiation pathway. The specific mechanism of action would be that the level of circRNA-circHmbox1 can be significantly reduced in TNF-a-induced osteoclast formation in vivo and in vitro. CircHmbox1 could inhibit RANKL-induced osteoclasts differentiation primarily by binding to microRNA-1247-5p. TNF-α decreased osteoblasts differentiation by exosomal with low circHmbox1 expression from osteoclasts. Mechanistic studies presented that microRNA-1247-5p regulated osteoclasts differentiation and osteoblasts differentiation by targeting Bcl6. These results confirmed that circHmbox1-targeting miR-1247-5p was engaged in the regulation of bone metabolisms by TNF-α in PMOP.^[14]^ Since TNF is a down-regulated gene in our differential gene screening, osteoclasts are active in differentiation, consistent with our research results. However, the specific expression of key genes in tissues reflected that TNF is highly expressed in osteoclasts. This lays a molecular foundation and provides a target site for further experimental research.

The incidence of metabolic syndrome is higher in TS patients. KEGG pathway suggested that genes were involved in the alanine metabolic pathway including DPYS. Dihydropyrimidinase gene (DPYS) was analyzed. Dihydropyrimidinase (DHP) was the second enzyme in the pyrimidine degradation pathway, which catalyzed the ring-opening of 5,6-dihydrouracil and 5,6-dihydrothymine to N-carbamoyl-β-alanine and N-carbamoyl-β-aminobutyric acid, respectively 2 patients with complete DHP deficiency were reported, both of whom were heterozygotes with missense mutation 1078T>C(W360R) in exon 6 and new missense mutation 1235G>T(R412M) in exon 7.^[15]^ Our DEG analysis revealed that DPYS is down-regulated in TS patients, resulting in lower alanine levels than normal people. The previous 2 latest research results also support our conclusion.^[16,17]^ The risk of autoimmune diseases in TS patients is around twice that of the general female population, and the spectrum covers IBD and celiac disease.^[18]^ This autoimmune disease may be correlated with an MHC locus on the long arm of the X chromosome, and the deletion of this region may cause insufficient immune regulation.^[19]^

The KEGG analysis of up-regulated genes demonstrated that these genes also participated in the process of *H pylori* infection. Previous reports have suggested a correlation between *H pylori* infection and idiopathic short stature. The positive rate of *H* *pylori* antibody in the idiopathic short group is significantly higher than that in the control group.^[20]^ Our study revealed the *H pylori* infection pathway in the selected key genes. This reflected that it might be related to TS’s short stature to some extent. The mechanism may be induced by the molecular similarity between *H pylori* and a peptide substance, which leads these antibodies to recognize *H pylori* and open the trigger mechanism of microbial antigen in the gastrointestinal tract, contributing to influencing the regulation of peptides produced in the digestive tract, adipose tissue, and brain on growth hormone secretion and food intake.^[21]^ At present, there is no molecular research on *H pylori* and TS. In our research results, 4 genes enriched in the KEGG pathway (ATP6V1A, MAPK9, ATP6AP1, CHUK) may be the new direction of related research.

There are few reports on hepatitis in the TS population. Calanchini et al^[22]^ evaluated the liver function of 125 TS patients. The results suggested that γ-glutamyltransferase (GGT) accounted for a relatively high proportion (about 88.7%), and the increase of LFTs (about 49.6%) was more essential because it might develop into severe liver disease, such as viral hepatitis, liver fibrosis, and liver cirrhosis.^[5]^ Additionally, a 5-year followup study reported that pathological anhydrase elevation is very common in TS women, and about 36% of patients in the followup population exhibited anhydrase elevation.^[23]^ However, this study demonstrated that the increase in such anhydrase was not related to viral hepatitis. Meanwhile, some reports suggested that diseases with abnormal neurodevelopment may lead to hepatitis.^[24]^ We discovered crucial genes involved in hepatitis formation in KEGG analysis of down-regulated genes. Since the relationship between TS patients and hepatitis is not clear, other prospective studies may be required for further verification.

Besides, further network protein analysis was performed on the differential genes to understand the interaction between proteins. The essential protein pairs were screened to select 10 hub genes. Among them, HMG20B, as a DNA binding gene, has been verified to play the role of erythroid differentiation inhibitor by downregulating differentiation-related genes (such as Hrasls3) and activating differentiation inhibitors (such as Gfi1b) in mice.^[25]^ Therefore, HMG20B may be engaged in the clinical manifestation of anemia in TS patients. Our analysis of the specific expression of key genes in tissues implied that HMG20B is highly expressed in osteoclasts.

Although the expression of hub genes was discovered, this study still has limitations. First, the database GSE58435 contains only 5 TS patients, and the sample size is small. Thus, the screened differential genes cannot be completely representative. Additionally, there are few studies on TS. When searching in the GEO database, only this database is an amniotic fluid sample. Hence, the stability of the results cannot be guaranteed by combining them with other data. Due to the lack of animal and human studies on TS at present, the screened differential genes cannot be verified correspondingly. As a result, some of our conclusions are not supported by evidence. The advantages of this study are described as follows. First, we found the possible influence of pathways such as *H pylori* that have not been proposed in previous studies on the height of Turner patients, and we included GSE46687 data set to verify hub genes. This can partly explain the stability of the current results. Considering that different sample sources or different included populations may lead to different results, the up-regulated genes of HIST1H2BA, TRIM71, and HIST1H2BB screened from the GSE58435 data set are down-regulated genes in the GSE46687 data set.

In conclusion, 733 DEGs and 10 hub genes were identified. They are new candidate targets in TS to understand the pathogenesis and progression mechanism, and may help to recognize the syndrome and open ways to new forms of treatment, so as to obtain a better prognosis. Although differential genes all play corresponding roles in the pathogenesis of Turner syndrome, the related hub genes of the common phenotype were detected in our analysis. This provides an imperative research basis for related clinical research and a new direction for further treatment more accurately. However, research at the molecule level of TS is up to now very limited, specifically more clinical research on the molecule level is wanted to be able to eventually relieve symptoms in TS.

## Author contributions

Tiantian Cheng performed the data analysis and wrote this manuscript. Xiaoli Li sorted out the data. Jinhu Chen conceived and designed the experiments. Linlin Yang and Jing Liu revised the manuscript. Guangyao Song and Huijuan Ma performed project coordination and supervised the project. All authors have seen and approved the final manuscript.

**Conceptualization:** Jinhu Chen, Huijuan Ma.

**Data curation:** Tiantian Cheng.

**Formal analysis:** Xiaoli Li.

**Methodology:** Linlin Yang.

**Supervision:** Jing Liu.

**Validation:** Xiaoli Li.

**Visualization:** Tiantian Cheng.

**Writing - original draft:** Tiantian Cheng.

**Writing - review & editing:** Guangyao Song, Huijuan Ma.
